# Gut microbiota dysbiosis contributes to the development of chronic obstructive pulmonary disease

**DOI:** 10.1186/s12931-021-01872-z

**Published:** 2021-10-25

**Authors:** Naijian Li, Zhouli Dai, Zhang Wang, Zhishan Deng, Jiahuan Zhang, Jinding Pu, Weitao Cao, Tianhui Pan, Yumin Zhou, Zhaowei Yang, Jing Li, Bing Li, Pixin Ran

**Affiliations:** 1 Department of Allergy and Clinical Immunology, State Key Laboratory of Respiratory Disease, National Clinical Research Center for Respiratory Disease, Guangzhou Institute of Respiratory Health, The First Affiliated Hospital of Guangzhou Medical University, 151 Yanjiang Road, Guangzhou, Guangdong People’s Republic of China; 2 College of Medicine, Lishui University, Lishui, Zhejiang People’s Republic of China; 3 Institute of Ecological Science, School of Life Science, South China Normal University, Guangzhou, Guangdong People’s Republic of China; 4 The GMU-GIBH Joint School of Life Sciences, Guangzhou Medical University, Guangzhou, Guangdong People’s Republic of China

**Keywords:** COPD, Gut microbiome, Fecal transplant, Mice, Lung inflammation

## Abstract

**Background:**

Dysbiosis of the gut microbiome is involved in the pathogenesis of various diseases, but the contribution of gut microbes to the progression of chronic obstructive pulmonary disease (COPD) is still poorly understood.

**Methods:**

We carried out 16S rRNA gene sequencing and short-chain fatty acid analyses in stool samples from a cohort of 73 healthy controls, 67 patients with COPD of GOLD stages I and II severity, and 32 patients with COPD of GOLD stages III and IV severity. Fecal microbiota from the three groups were then inoculated into recipient mice for a total of 14 times in 28 days to induce pulmonary changes. Furthermore, fecal microbiota from the three groups were inoculated into mice exposed to smoke from biomass fuel to induce COPD-like changes.

**Results:**

We observed that the gut microbiome of COPD patients varied from that of healthy controls and was characterized by a distinct overall microbial diversity and composition, a *Prevotella*-dominated gut enterotype and lower levels of short-chain fatty acids. After 28 days of fecal transplantation from COPD patients, recipient mice exhibited elevated lung inflammation. Moreover, when mice were under both fecal transplantation and biomass fuel smoke exposure for a total of 20 weeks, accelerated declines in lung function, severe emphysematous changes, airway remodeling and mucus hypersecretion were observed.

**Conclusion:**

These data demonstrate that altered gut microbiota in COPD patients is associated with disease progression in mice model.

**Supplementary Information:**

The online version contains supplementary material available at 10.1186/s12931-021-01872-z.

## Background

Chronic obstructive pulmonary disease (COPD) is a worldwide public health concern and a leading cause of morbidity and mortality [1, 2]. Currently, bronchodilator and anti-inflammatory therapies are still the mainstay of pharmacological treatment in COPD [3]. Although progress has been made in the treatment of symptoms and prevention of acute exacerbations, few advances have been achieved in ameliorating disease progression or affecting mortality [2, 4]. COPD is a complex disease with multiple sub-phenotypes that affects not only the lungs but also the cardiovascular, gastrointestinal, and immune systems, and the underlying mechanisms are not completely understood [5–7].

The role of the microbiome, and the gut microbiome in particular, has drawn considerable attention in human health and disease. Emerging evidence has revealed the role of gut microbiota dysbiosis in various chronic human diseases [8, 9]. Moreover, by fecal microbiota transplantation (FMT) experiments in animals and in humans, the causal relationships between the gut microbiota and the pathogenesis of multiple diseases in humans have been demonstrated. For example, gut microbiota dysbiosis contributes to the development of hypertension, and the short-chain fatty acid (SCFA) propionate, which is produced from dietary fiber by gut bacteria, has been shown to ameliorate hypertensive end-organ damage [10, 11]. Alteration of the gut microbiota by the antibiotic agent azithromycin reduces airway inflammation in allergic asthmatic patients [12]. However, little is known about the involvement of the gut microbiota in COPD. Although primarily considered a respiratory disease, COPD commonly co-occurs with chronic gastrointestinal tract diseases [7, 13]. Recent studies have linked changes in the gut microbial composition and function to disease development in the lungs, but knowledge of the gut-lung axis in COPD is scarce [14, 15]. The features of the gut microbiota in patients with COPD remain to be determined and the contribution of gut microbes to disease progression is still poorly understood.

In the present study, we performed 16S ribosomal RNA sequencing of stool samples from COPD patients and healthy controls, described the features of the gut microbiota, and analyzed their SCFA profiles. Next, we performed fecal microbiota transplantation into mice, demonstrating the role of disordered gut microbiome in COPD.

## Methods

### Study design

**Fig. 1:**
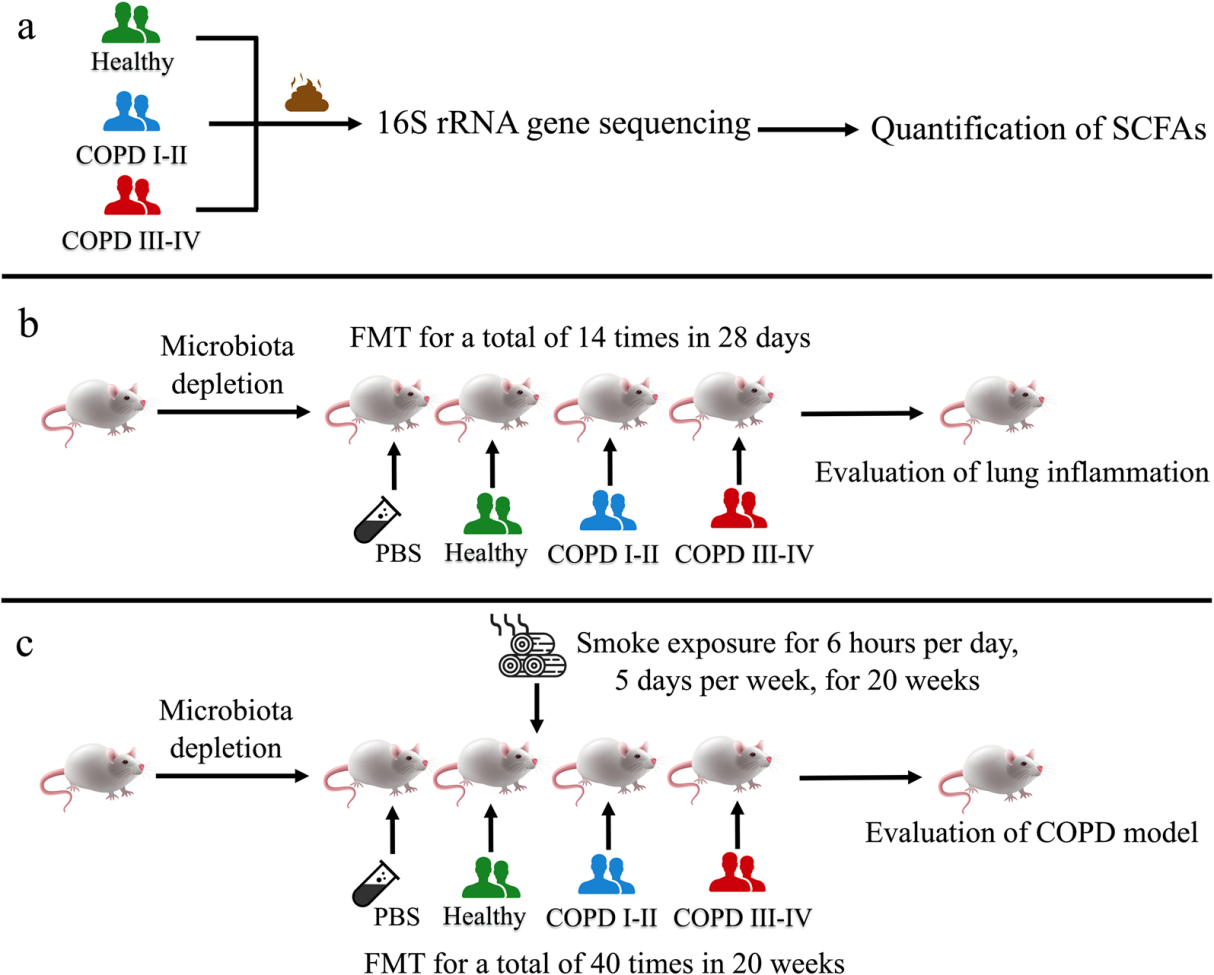
Schematic overview of the study workflow. **a** Healthy individuals (n = 73) and COPD patients (n = 99) were recruited for the study. Stool samples were subjected to 16S ribosomal RNA sequencing and short-chain fatty acid (SCFA) profiling. **b** Fecal microbiota from healthy individuals and from COPD patients were pooled and inoculated into mice by fecal microbiome transplantation for inflammatory phenotyping. **c** During the biomass fuel exposure, the fecal microbiota transplantation was performed

To investigate the features of the gut microbiota in COPD patients and the contribution of the gut-lung axis in COPD, we designed a systematic and reproducible workflow (Fig. 1a–c). All subjects in the present study were recruited from the 135 Key Research and Development Program (No._2016YFC1304101), which is a population-based, cross-sectional, multicenter survey of COPD conducted in China. In our previous COPD studies in this cohort [16–18], we obtained a representative sample of the COPD population in Wengyuan and Guangzhou, and the healthy group comes from the same village or residential area. All patients were residents of Wengyuan and Guangzhou, which are approximately 130 km apart and characterized by similar lifestyles and eating habits. All recruited patients and healthy subjects provided written informed consent before stool donation.

### Study cohort and patient characteristics

All participants underwent pulmonary function measurements to diagnose COPD according to GOLD guidelines [3]. Spirometry data were obtained as previously described [18]. The participants were classified by pulmonary function as described in GOLD guidelines. The final cohort was composed of 73 healthy controls (FEV_1_/FVC ratio ≥ 70% and FEV_1_ ≥ 80%), 67 patients with COPD GOLD severity stages I and II (FEV_1_/FVC ratio < 70% and FEV_1_ 50–80%), and 32 patients with COPD severity stages III and IV (FEV_1_/FVC ratio < 70% and FEV_1_ < 50%).

All individuals underwent medical tests prior to stool sample collection, including chest X-ray, electrocardiography, abdominal ultrasound, and blood, urine, and stool tests. Subjects were required to meet the following criteria to qualify for the study: (1) Able and willing to provide written informed consent and medical records from the preceding year. (2) Male aged 40–80 years. (3) Diagnosed with COPD more than 1 year prior to the study. Subjects who met any of the following criteria prior to enrollment were excluded from the study: (1) Treatment with systemic (e.g., oral, intravenous, or intramuscular) corticosteroids within the preceding 8 weeks. (2) History of cystic fibrosis, asthma, and/or other clinically significant lung disease other than COPD. (3) A diagnosis of cancer, heart failure, hypertension, diabetes, infectious diseases, renal or liver dysfunction, gastrointestinal disease, or treatment with antibiotics (inclusive of macrolide antibiotics) within the preceding 8 weeks. All clinical information was collected according to standard procedures by the State Key Laboratory of Respiratory Disease of Guangzhou Medical University (Guangzhou, China).

### Stool sample collection and sequencing

Fresh stool from donors was collected in the morning. The consistency of each sample was graded according to the Bristol Stool Form Scale, and only sample types 2–5 were included [19]. Stool samples in sterile containers were snap-frozen in dry ice and stored after arrival at the research laboratory in − 80 °C freezers until processing. Total bacterial DNA extraction of stool was carried out on 100 mg of sample using the HiPure Stool DNA kits (Magen BioSciences, Waltham, MA, USA) in accordance with the manufacturer’s instructions. The extracted DNA from each sample was used as a template to amplify the V3–V4 region of 16S rRNA genes using PCR. 16S rRNA gene amplification, in vitro transcription and labeling, and hybridization were conducted using the Illumina 16S Metagenomic Sequencing Library preparation guide [20]. 16S amplicon PCR forward primer 5′-(TCG TCG GCA GCG TCA GAT GTG TAT AAG AGA CAG CCT ACG GGN GGC WGC AG)-3′ and 16S amplicon PCR reverse primer 5′ -(GTC TCG TGG GCT CGG AGA TGT GTA TAA GAG ACA GGA CTA CHV GGG TAT CTA ATC C)-3′ were used [21]. All libraries were sequenced by using an Illumina MiSeq PE250 platform (San Diego, CA, USA) at the RiboBio Genome Center (Guangzhou, China).

### Gut microbiota analysis

16S rRNA gene sequence analysis, including raw sequence filtering and taxonomic classification, was performed as described previously [22]. Briefly, the raw sequencing data were filtered for quality (Q30) and joined by FLASH ( http://ccb.jhu.edu/software/FLASH/) [23]. Sequences that contained read lengths shorter than 200 bp were removed. The QIIME (Quantitative Insights into Microbial Ecology, v1.9.1, http://qiime.org/) software pipeline was used to cluster high-quality reads into operational taxonomic units (OTUs) at the 97% identity level. OTU search was performed using the GreenGenes 13.8 database. The α-diversity indices (Chao1 index) was calculated by QIIME. The bar diagram of alpha diversity indices and relative abundance were drawn using GraphPad Prism 8 software (GraphPad Software Inc., San Diego, CA, USA).

Enterotyping was run with a reference-based online tool (http://enterotypes.org) as indicated by Costea et al. [24]. Only genera with an average relative abundance ≥ 10^−4^ and that appeared in at least 50% of samples in each group were considered in the analysis. Pearson’s Chi-squared test was used for testing enterotypes between clinical groups. Significance of community dissimilarity based on Bray–Curtis dissimilarity matrices was tested using a permutational multivariate analysis of variance (PERMANOVA) function (adonis) within the R package vegan.

### Quantification of SCFAs in stool samples

Seven SCFAs (acetic, propionic, butyric, isobutyric, valeric, isovaleric, and caproic acids) were measured in the stool samples by high-performance gas chromatography (Agilent 6890N; Agilent Technologies, Santa Clara, CA, USA) with an autosampler and a flame ionization detector according to the manufacturer’s guidelines.

## Animal experiments

### Mice

Specific pathogen-free male C57BL/6 mice were purchased from Guangzhou University of Chinese Medicine (Guangzhou, China). The mice were housed five to a cage. All experiments were conducted with mice with 8–10 weeks of age. Temperature and relative humidity in the animal facility were controlled at 23 ± 2 °C and at 40–70%, respectively. Lighting was artificial with a sequence of 12 h of light (06:00–18:00) and 12 h of darkness. Rodent food pellets and water were sterilized and provided ad libitum. The Animal Medical Center of Guangzhou Medical University reviewed and approved all experiments (identification number: GY2018-084).

### Microbiota depletion and fecal transplantation

To deplete the gut microbiota, mice were provided with broad-spectrum antibiotics (ampicillin 1 g/L; neomycin sulfate 1 g/L; metronidazole 1 g/L; vancomycin 0.5 g/L, all purchased from Sigma-Aldrich, St. Louis, MO, USA) in drinking water for 3 weeks as previously described [25]. Treatment with broad-spectrum antibiotics in drinking water was stopped 2 days before fecal microbiota transplantation. 1 g fecal matter from each human group was mixed and then resuspended in 4 mL of phosphate-buffered saline and homogenized. The homogenate was centrifuged at 200×*g* for 10 min at 4 °C, the supernatant was collected and stored at − 80 °C for subsequent use. Enumeration of inoculum from healthy group, COPD I-II group and COPD III-IV group in 1 mL of initial suspension were performed according to plate count methods [26].

### Gut microbiota-induced murine model of pulmonary inflammation

A total of 60 mice were randomly divided into four groups of 15: Phosphate-buffered saline (PBS) FMT group, healthy FMT group, COPD I–II patients FMT group, and COPD III–IV patients FMT group. After microbiota depletion, the fecal microbiota transplantation was performed by a single oral administration of 100 μL per mouse every other day, for a total of 14 times in 28 days. The mice received fecal transplants from healthy individuals, COPD I–II subjects, or COPD III–IV subjects, and PBS as control.

### Fecal transplantation experiment in biomass smoke-induced murine model of COPD

Similar to the previous, a total of 60 mice were also randomly divided into four groups of 15: PBS FMT group, healthy FMT group, COPD I–II patients FMT group, and COPD III–IV patients FMT group. After microbiota depletion, all mice were exposed to smoke produced by smoldering China fir sawdust (40 g/exposure) for two 3-h periods, 5 days per week, for 20 weeks in an inhalation chamber (model INH-WB_NOE (R/M)_CAP (PM2.5)_CS_SP; TSE Systems, Bad Homburg, Germany) [27]. Particulate matter mass concentrations, particle size distributions, and gas concentrations (oxygen, carbon monoxide, nitrogen oxides, and sulfur dioxide) were monitored by a DustTrak II aerosol monitor 8530 (TSI, Shoreview, MN, USA) and a Testo 340 portable flue gas analyzer (Testo, Lenzkirch, Germany) in the exposure rooms. During the biomass smoke exposure, the fecal microbiota transplantation was performed by a single oral administration of 100 μL per mouse twice a week, for a total of 40 times in 20 weeks.

### Measurement of lung function

Spirometry data were obtained as previously described using a Forced Pulmonary Maneuver System (Buxco Research Systems, Wilmington, NC, USA) [28]. Mice were sedated with 3% pentobarbital (1 mL/Kg), and were tracheostomized and intubated, then the mice placed supine in the body chamber and connected to the system. The depth of anesthesia was maintained at a light surgical plane for the duration of testing, and the dose could be adjusted as necessary. The Cchord (chord compliance, between 0 and 10 cm H_2_0), FEV_20_ (forced expiratory volume in 20 s), FRC (functional residual capacity), PEF (peak expiratory flow), MMEF (mean mid expiratory flow) and minute ventilation volume were measure within 10 min. At least three acceptable maneuvers for each test of every mice were conducted to obtain a reliable mean spirometry data.

### Bronchoalveolar lavage fluid differential cell count and biomarker analysis

Mice were sacrificed by CO_2_ and lung tissue and blood samples were collected. Whole lungs were cyclically inflated and deflated with 1 mL of phosphate-buffered saline (Gibco-Thermo Fisher Scientific, Waltham, MA, USA) three times. Cells were isolated by centrifugation at 300×*g* for 10 min at 4 °C and stained with Diff-Quik stain (Baso Diagnostics, Zhuhai, China). Differential cell counts were assessed from 400 cells counted on each slide. Plasma cytokine levels were assayed using Luminex xMap and a commercially available mouse cytokine 6-plex panel (Bio-Rad, Hercules, CA, USA) according to the manufacturer’s guidelines and measured on a Bio-Plex 200 Platform.

### Flow cytometry

Flow cytometry immunophenotyping was performed on T cells and B cells in whole blood. Peripheral blood mononuclear cells were isolated from sodium heparin-treated venous blood samples by Ficoll-Hypaque density gradient and centrifugation at 1000×*g* at room temperature for 20 min. We blocked the Fc receptors by incubating cells first with anti-CD16/32 antibodies for 15 min on ice. Cells were stained with the following antibodies for 30 min at room temperature: BB600-conjugated anti-mouse CD3, APC-conjugated anti-mouse CD19, BV421-conjugated anti-mouse CD8, and FITC-conjugated anti-mouse CD4 (BD Biosciences, Franklin lakes, NJ, USA). After washing, cells were fixed with fluorescence-activated cell sorting lysing solution (BD Biosciences). Appropriate isotype controls were used to determine the specificity of the staining. Flow cytometry data were acquired using FACSVerse (BD Biosciences) and analyzed with the FlowJo software (Tree Star, Inc., Ashland, OR, USA).

### Protein isolation and western blot assay

Lung tissues were homogenized on ice for analysis of MUC2, MUC5AC, the tight junction protein claudin 1, α smooth-muscle actin (a-SMA), matrix metalloproteinase 2 (MMP-2), and neutrophil elastase levels. Total proteins were extracted from 100 mg of lung tissues from each group, and concentrations were determined by the BCA protein assay. Thirty micrograms of total protein were loaded into each well and fractionated on a 10% SDS polyacrylamide gel. The housekeeping gene *β*-tubulin was used as an internal control to assess equal loading of total protein between wells. The bound antibodies were visualized using SuperSignal West Femto Maximum Sensitivity Substrate (Thermo Fisher Scientific). The abundance of target proteins was quantified by enhanced chemiluminescence.

### Pathology and immunohistochemistry

Lung tissues were fixed with 4% paraformaldehyde solution and embedded in paraffin using standard methods as described previously [29]. Consecutive sections (3–5 µm) were prepared, mounted on glass slides, and stained with hematoxylin and eosin. All slides were scanned and analyzed using an image analyzer platform (Leica, Wetzlar, Germany). Lung sections were stained with Alcian blue-periodic acid-Schiff (AB-PAS) using commercial kits (Sigma-Aldrich, St. Louis, MO, USA). The sections were incubated with primary antibodies against a-SMA, MMP-2, or MUC5AC (Abcam; Cambridge, UK). Quantitative analysis of IHC staining was performed using Image-Pro Plus 6.0 (Media Cybernetics, Bethesda, MD, USA). The alveolar destruction and the bronchial wall thickness were quantified as previously described [30]. Bronchial wall thickness was calculated as wall thickness = (total bronchial area − lumen area)/total bronchial area, and the alveolar enlargement and destruction were quantified by the mean linear intercept (Lm). Sectioning and staining were performed by the Pathology Center of the First Affiliated Hospital of Guangzhou Medical University.

### Statistical analysis

In the human cohort, microbiota data and SCFA levels were tested by ANOVA and the Kruskal–Wallis H test. The *p* values were corrected for multiple testing using the Bonferroni method. Analyses were adjusted by age and smoking history. Pearson’s χ^2^ test was used for testing enterotypes between clinical groups. Significance of community dissimilarity from Bray–Curtis dissimilarity matrices was tested using a permutational multivariate analysis of variance function (adonis) within the R package vegan. For animal experiments, comparisons were performed using ANOVA and *p* values were also corrected using the Bonferroni method. Statistical analysis was performed in SPSS version 24 (IBM SPSS, Armonk, NY, USA), and the corrected *p* < 0.05 was considered significant.

## Results

### Characteristics of the study population

A total of 172 Chinese participants were enrolled and divided into healthy controls (n = 73), COPD stage I–II (n = 67), and COPD stage III–IV (n = 32). Table 1 summarizes the characteristics of study participants. The subjects in the COPD group had lower body mass index, forced expiratory volume in 1 s, forced vital capacity, and the forced expiratory volume in 1 s to forced vital capacity ratio but higher smoking index and COPD assessment scores than subjects in the control group. No significant difference was observed in occupation among the three groups.

**Table 1:** Demographic characteristics of the study cohorts

Characteristics	Healthy (n = 73)	COPD I–II (n = 67)	COPD III–IV (n = 32)	*p* value
Age, years	59.7 ± 9.4	62.7 ± 6.7	61.5 ± 6.6	NS
BMI, kg/m^2^	24.6 ± 2.9	22.7 ± 3.6	20.8 ± 2.7	< 0.01^abc^
Smoking index (pack-yr)	18.3 ± 23.4	40.8 ± 28.8	42.8 ± 30.2	< 0.01^ab^
Smoking status, n (%)				< 0.001^ab^
Never smoker	38 (52.05%)	0	0	–
Current smoker	20 (27.40%)	48 (71.64%)	17 (53.10%)	< 0.001
Ex-smoker	15 (20.55%)	19 (28.36%)	15 (46.90%)	< 0.001
Occupation, n (%)				
Manual work in industry	14 (19.18%)	12 (17.91%)	3 (9.38%)	0.809
Manual work in service	10 (13.70%)	11 (16.42%)	3 (9.38%)	
Farmer	40 (54.79%)	33 (49.25%)	20 (62.50%)	
Professionals	4 (5.48%)	7 (10.45%)	4 (12.50%)	
Others	5 (6.85%)	4 (5.97%)	2 (6.24%)	
CAT score	–	8 (3,11)	18 (14,24)	< 0.001^c^
MMRC score	–	1 (0,1)	3 (2,3)	< 0.001^c^
FEV_1_ (L)	2.80 ± 0.56	2.07 ± 0.52	0.96 ± 0.34	< 0.001^abc^
FEV_1_%	95.07 ± 14.53	73.88 ± 16.72	34.35 ± 9.91	< 0.001^abc^
FVC (L)	3.66 ± 0.68	3.44 ± 0.65	2.31 ± 0.53	< 0.001^bc^
FVC%	99.73 ± 13.90	97.13 ± 15.44	66.53 ± 12.63	< 0.001^bc^
FEV_1_/FVC%	76.22 ± 6.32	60.09 ± 8.64	42.82 ± 10.95	< 0.001^abc^

Current smokers were defined as those who smoked more than five cigarettes per day during the past month; ex-smokers were defined as those who had smoked more than five cigarettes previously and had stopped for > 1 month; nonsmokers were defined as those who had never smoked or patients who smoked no more than five cigarettes per day at the time of the or previously. Data are shown as means ± SD or median (interquartile range). Differences in continuous variables were evaluated using ANOVA, and *p* values were corrected using the Bonferroni method

FEV_1_, forced expiratory volume in 1 s; *FVC* forced vital capacity, *BMI* body mass index, *NS* no significant difference, *CAT* COPD assessment test, *MMRC* modified Medical Research Council

^a^*p* < 0.05 for equality between the chronic obstructive pulmonary disease (COPD) I–II group and healthy subjects

^b^*p* < 0.05 for equality between the COPD III–IV group and healthy subjects

^c^*p* < 0.05 for equality between the COPD III–IV and COPD I–II groups.

### Altered gut microbial diversity and composition in COPD patients

**Fig. 2:**
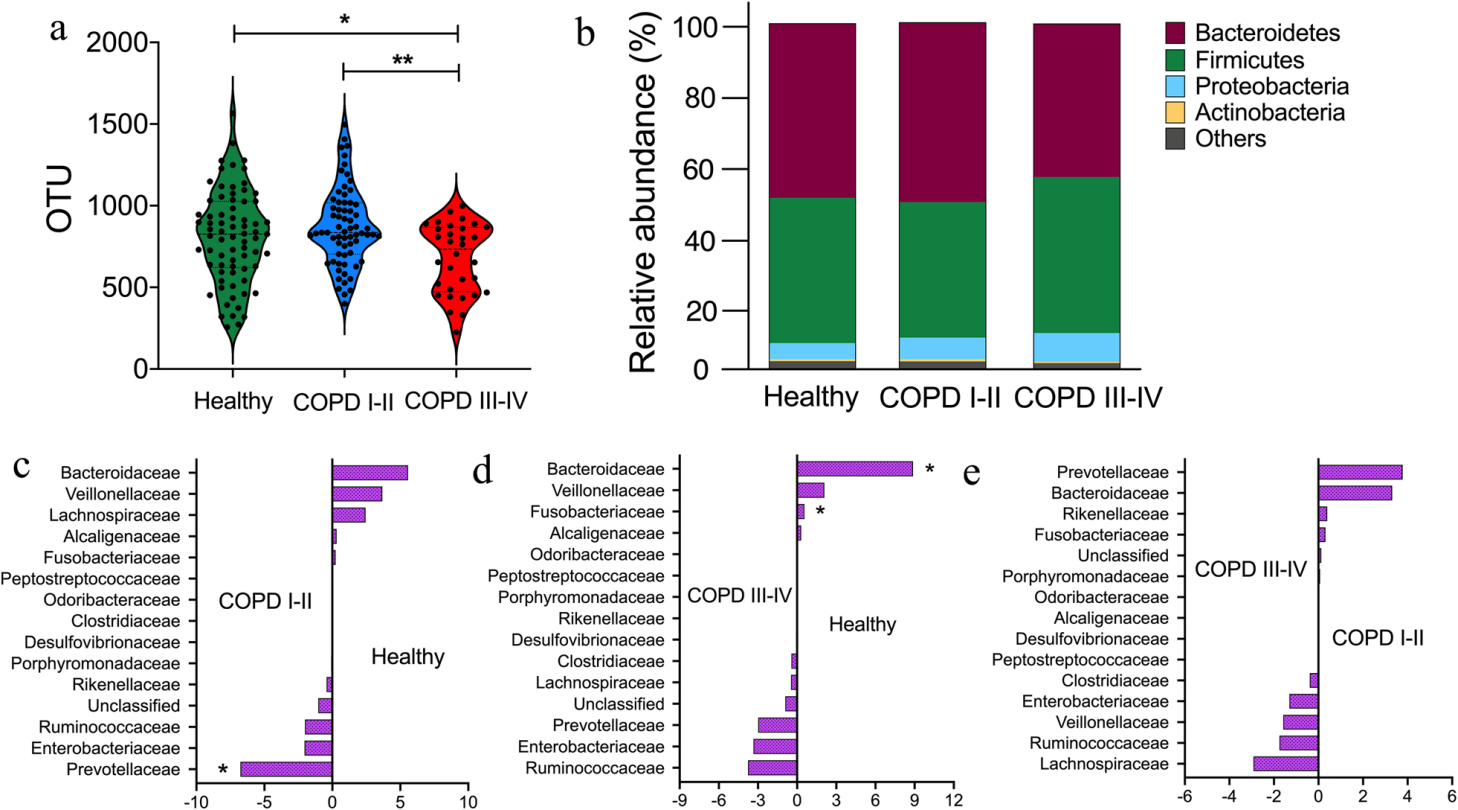
Gut microbial diversity differs between healthy controls and chronic obstructive pulmonary disease (COPD) patients. **a** Comparison of the operational taxonomic units (OTUs) in the three subject groups. **b** Relative proportions of bacterial phyla in the three subject groups. **c**–**e** Comparisons of the abundance of major bacterial families in the three subject groups. The x axis denotes relative abundance (%). The values on the x-axis are expressed as delta between the median relative abundance (%) of the right-side group and the median relative abundance (%) of the group on the left. Healthy, n = 73; COPD I–II, n = 67; COPD III–IV, n = 32. Significance was determined by ANOVA and by Kruskal–Wallis test, and *p* values were corrected using the Bonferroni method. *p < 0.05, **p < 0.01

To assess whether gut microbial changes are associated with COPD severity, we performed 16S rRNA gene sequencing of fecal samples. Comparison between the three groups showed that intra-individual diversity, as measured by the number of operational taxonomic units (OTU), was significantly lower in the COPD III–IV group (*p* = 0.049 vs. healthy subjects and *p* = 0.009 vs. COPD I–II group, Fig. 2a). At the phylum level, all gut microbiota samples from the healthy group, COPD I–II group, and COPD III–IV group contained four major bacterial phyla: Bacteroidetes, Firmicutes, Proteobacteria, and Actinobacteria. The first three phyla accounted for over 96% of the total sequences in all three groups. The COPD III–IV group had a lower relative abundance of Bacteroidetes (*p* = 0.040 vs. healthy group; *p* = 0.007 vs. COPD I–II group) but a higher abundance of Firmicutes (*p* = 0.041 vs. COPD I–II group; Fig. 2b and Additional file 4: Table S1). The sequences represented in the four phyla were mainly distributed into 15 bacterial families, which accounted for over 95% of the total sequences in all three groups. Three bacterial families differed in their relative abundance in samples from the three groups (Fig. 2c–e and Additional file 4: Table S2). A higher abundance of *Prevotellaceae* was observed in the COPD I–II group (*p* = 0.046 vs. healthy controls). Compared with the healthy controls, COPD III–IV subjects exhibited a lower abundance of *Bacteroidaceae* (*p* = 0.034) and *Fusobacteriaceae* (*p* = 0.002). There were no significant differences between COPD I–II and COPD III–IV subjects.

**Fig. 3:**
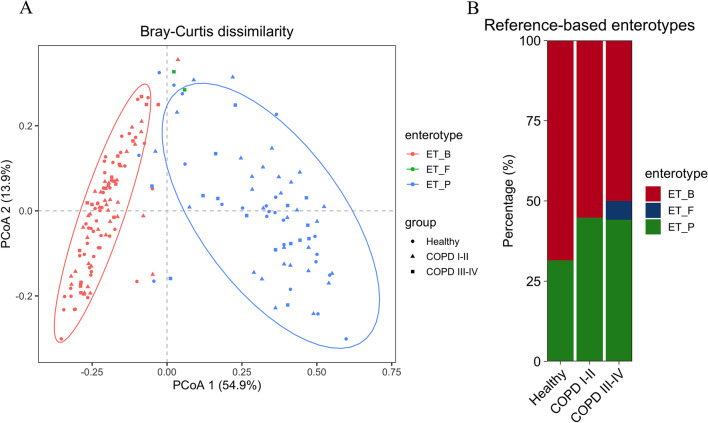
Gut enterotypes shift in human adults with chronic obstructive pulmonary disease (COPD). **a** Enterotypes in clinical groups. A total of 172 samples (Healthy, n = 73; COPD I-II, n = 67; COPD III-IV, n = 32) are clustered into ET_P (‘Prevotella’ enterotype, blue) and ET_B (‘Bacteroides’ enterotype, red) by PCoA of Bray–Curtis dissimilarity at the genus level. **b** Percentage of individuals from each group characterized by ET_P (green), ET_B (red), and ET_F (‘Firmicutes’ enterotypes, blue). The major contributors in the two enterotypes are Prevotella and Bacteroides, respectively. Each dot corresponds to a sample

In *β*-diversity analysis, the variance of the 172 samples was largely explained by the first principal component (54.9%), corresponding to differing enterotypes but not clinical groups (Fig. 3a). Most healthy controls (68.5%) were clustered in the ‘Bacteroides’ enterotype (ET_B), while approximately 45% of subjects in each COPD patient group were clustered in the ‘Prevotella’ enterotype (χ^2^ test, *p* = 0.072, healthy vs. COPD I–II subjects; *p* = 0.149, healthy vs. COPD III–IV subjects; *p* = 0.091, healthy vs. all COPD subjects; Fig. 3b). Permutational multivariate analysis of variance of Bray–Curtis dissimilarity demonstrated statistically significant differences in gut microbiota composition between healthy controls and all COPD subjects (*p* = 0.011).

### SCFA profiles

**Fig. 4:**
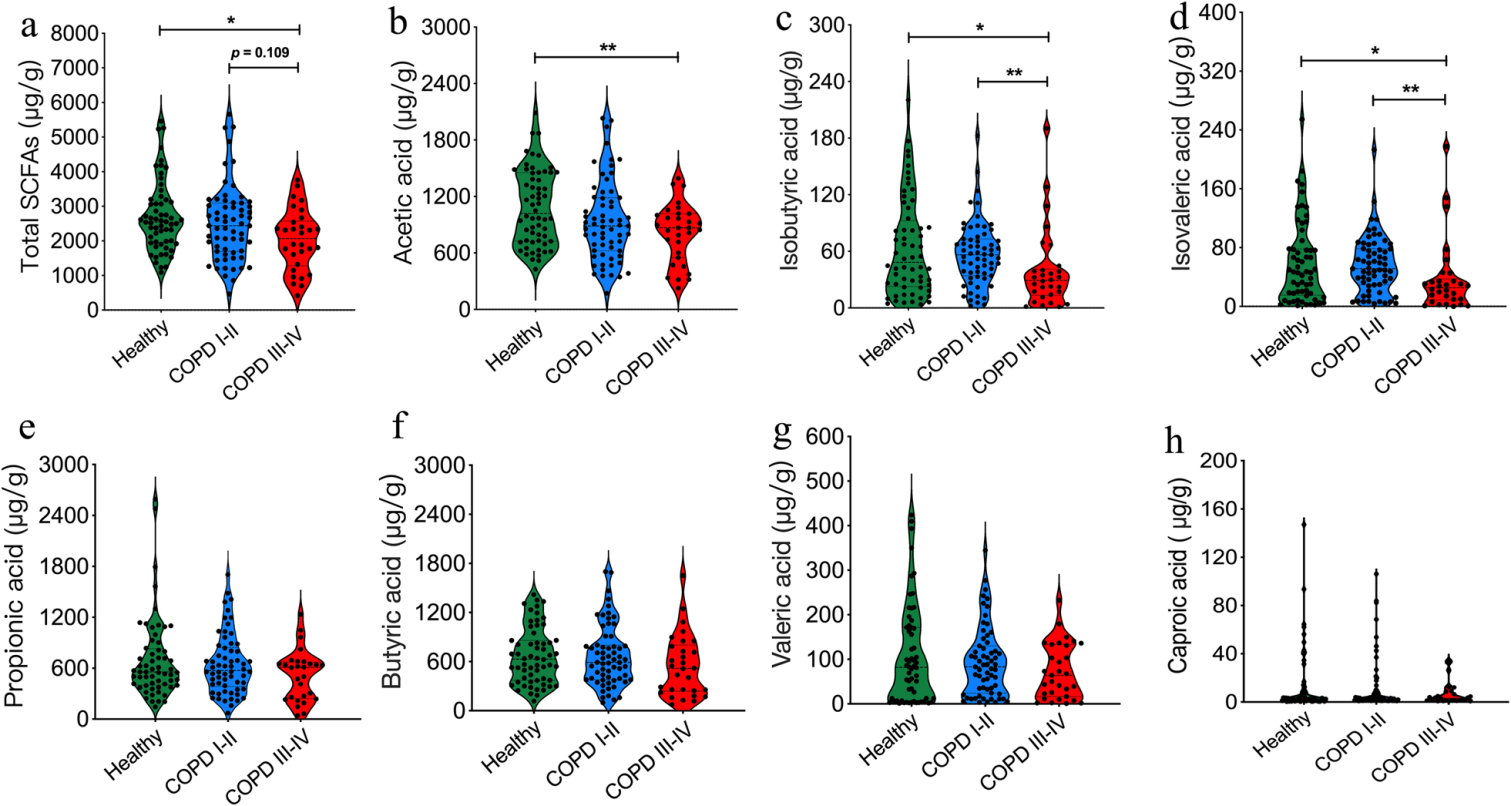
Short-chain fatty acid levels in fecal samples from healthy, chronic obstructive pulmonary disease (COPD) I–II subjects, and COPD III–IV subjects. **a** The levels of total short-chain fatty acids were significantly lower in COPD III–IV subjects. **b**–**d** Acetic acid, isobutyric acid, and isovaleric acid levels were also lower in COPD III–IV subjects. **e**–**h** Reductions in the levels of propionic acid, butyric acid, valeric acid, and caproic acid were observed in COPD patients, although this trend was not statistically significant. Significance was determined by the ANOVA test, and *p* values were corrected using the Bonferroni method. Each dot corresponds to a sample. Healthy, n = 60; COPD I–II, n = 63; COPD III–IV, n = 30. *p < 0.05, **p < 0.01

We compared the levels of intestinal SCFAs in stool samples from 60 healthy controls, 63 COPD I–II patients, and 30 COPD III–IV patients by gas chromatography. All seven SCFAs were detected in all stool samples. Acetic, propionic, and butyric acids accounted for over 89% of total SCFAs in all three groups, with acetic acid being most abundant (37.4–41.3%). Total SCFAs levels were significantly lower in the COPD III–IV group than in healthy (*p* = 0.012) and COPD I–II (*p* = 0.109) subjects (Fig. 4a). Moreover, a significant decrease in acetic acid levels was observed in the stool samples of COPD III–IV subjects (*p* = 0.007, Fig. 4b). Levels of isobutyric acid (*p* = 0.016 vs. healthy group; *p* = 0.007 vs. COPD I–II group) and isovaleric acid (*p* = 0.038 vs. healthy group; *p* = 0.006 vs. COPD I–II group) were also significantly lower in the COPD III–IV subjects (Fig. 4c, d). There was no significant difference in the levels of propionic acid, butyric acid, valeric acid, or caproic acid between groups (Fig. 4e–h). Overall, the severity of COPD was correlated with reductions in the levels of SCFAs.

### Intestinal microbiota from COPD patients induced lung inflammation and adaptive immune responses in mouse model

**Fig. 5:**
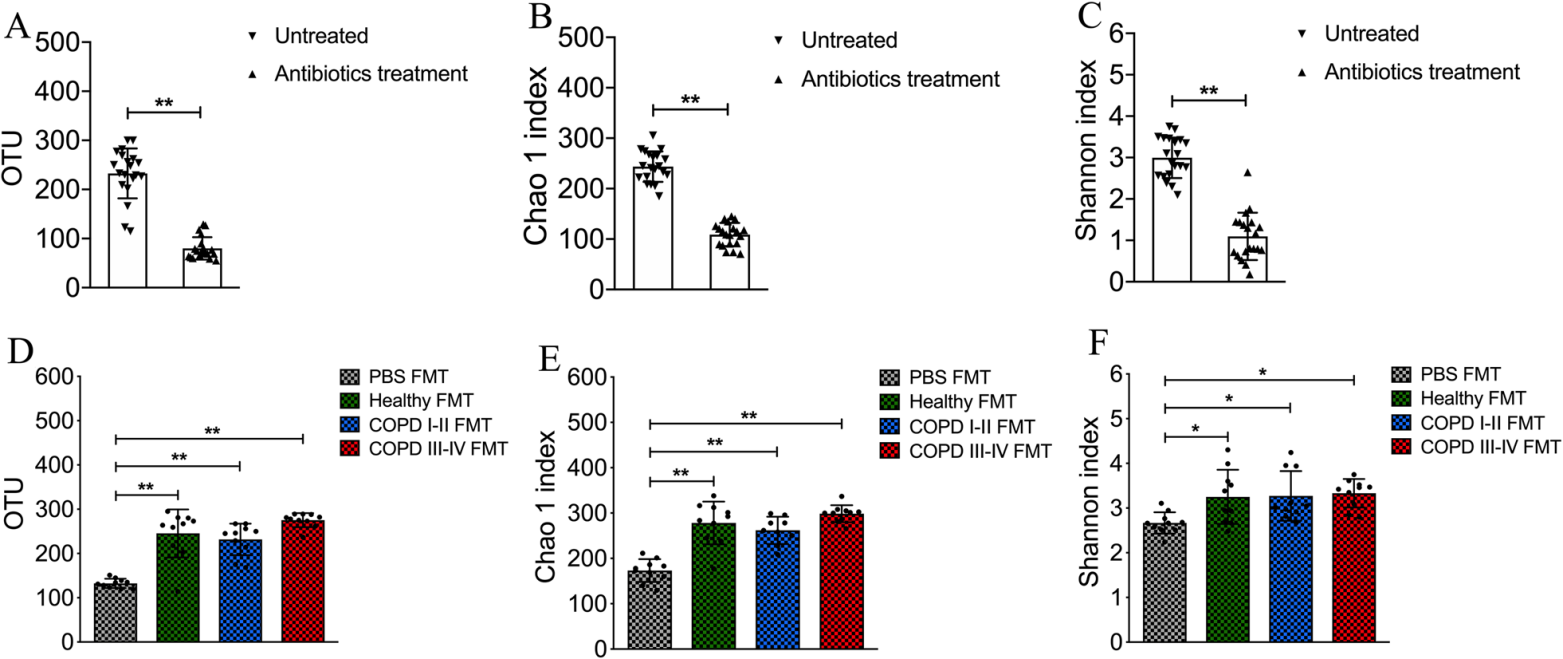
Microbial abundance and diversity in mice following antibiotics treatment and transplants of human fecal matter. **a**–**c** The gut bacterial abundance (operational taxonomic units, OTUs) and diversity (Chao1 index and Shannon index) in mice was significantly lower after treatment with broad-spectrum antibiotics in drinking water for 3 weeks, n = 20 mice. **d**–**f** The mice that received human fecal transplants over 28 days exhibited higher gut bacterial abundance and diversity than the PBS FMT group (no fecal transplants), n = 10 mice. *p < 0.05, **p < 0.01

**Fig. 6:**
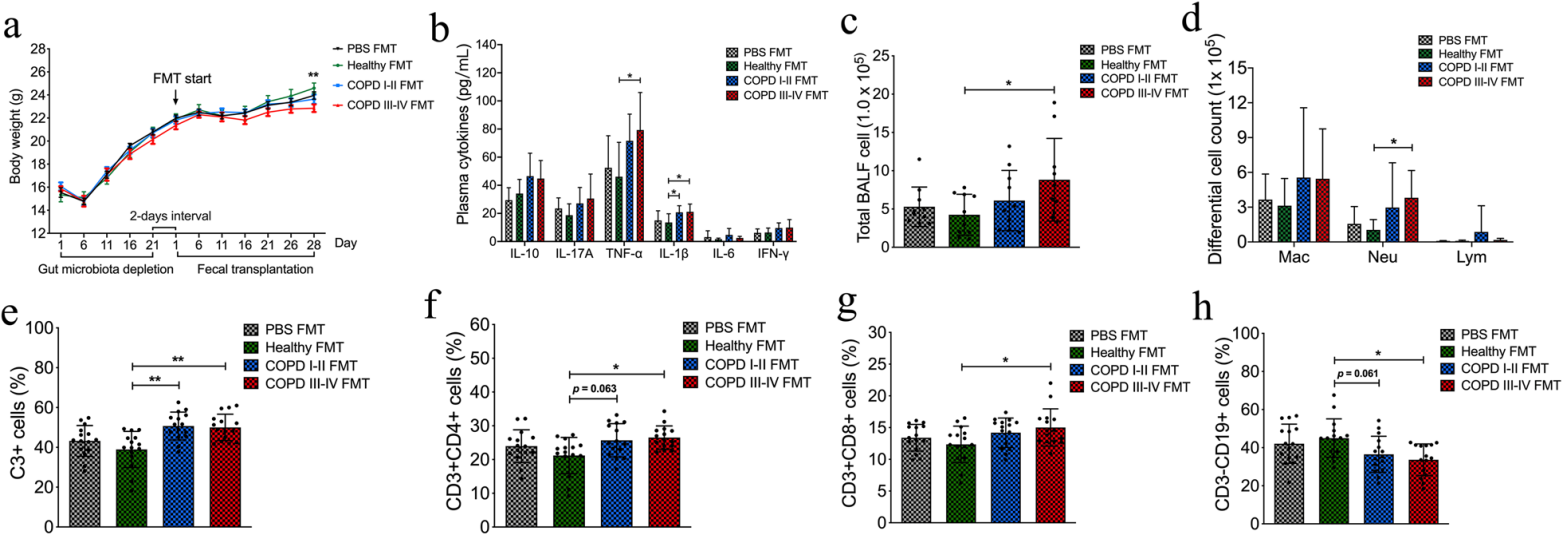
Intestinal microbiota from subjects with chronic obstructive pulmonary disease (COPD) induce pulmonary inflammation in recipient mice. **a** Intestinal microbiota from COPD III–IV patients reduced body weight in recipient mice. Values are means ± SEM, n = 15 mice. **b** Levels of interleukin (IL)-1β and tumor necrosis factor α in plasma increased significantly in mice that received fecal microbiota transplantation (FMT) from COPD III to IV patients. An upward trend was also observed for IL-6, IL-10, IL-17A, and interferon γ, although it did not reach statistical significance, n = 15 mice. **c**, **d** Total and differential leukocytes were counted in bronchoalveolar lavage fluid, n = 10 mice. **e** CD3 + T lymphocyte counts were higher in the peripheral blood of mouse recipients of fecal microbiota from COPD patients. **f**–**h **Increased counts of CD3 + CD4 + cells and CD3 + CD8 + cells but decreased counts of CD3-CD19 + cells were observed in mouse recipients of fecal microbiota from COPD patients, n = 15 mice. Significance was determined by ANOVA and *p* values were corrected using the Bonferroni method. Mac, macrophages; Neu, neutrophils; Lym, lymphocytes. *p < 0.05, **p < 0.01

After a 3-week antibiotics treatment, the gut bacterial abundance from mice was significantly lower (Fig. 5a–c). Compared with PBS FMT group, the mice that received human fecal transplants over 4 weeks exhibited higher bacterial diversity (Fig. 5d–f) and differed microbiota composition (Additional file 1: Fig. S1, Additional file 4: Table S2), confirming successful gut microbiota transfer. The viability of bacteria in the inoculum can be seen in Additional file 4: Table S3. A significant reduction in body weight was observed in mice that received microbiota from COPD III–IV patients (*p* < 0.01 vs. healthy controls, Fig. 6a) on day 28. Increased plasma cytokine levels were observed in the mouse recipients of fecal microbiota from COPD III–IV patients: levels of interleukin (IL) 1β (*p* = 0.034) and tumor necrosis factor α (*p* = 0.029) were significantly higher (Fig. 6b), and an upward trend was observed for IL-6, IL-10, IL-17A, and interferon γ, although these changes were not statistically significant. Mouse recipients of fecal microbiota from COPD III to IV subjects had higher total cell counts and the percentage of leukocytes in bronchoalveolar lavage fluid (*p* < 0.05, Fig. 6c, d), when compare to the healthy FMT group. Flow cytometry immunophenotyping was performed on T cells and B cells in whole blood. T and B lymphocyte sub-populations were defined as B lymphocytes (CD3 − CD19 + cells), auxiliary T lymphocytes (CD3 + CD4 + cells), and cytotoxic T lymphocytes (CD3 + CD8 + cells). CD3 + T lymphocyte counts were higher in the peripheral blood of mice that received fecal microbiota from COPD I to II patients (*p* < 0.01) and COPD III–IV patients (*p* < 0.01) than from healthy controls (Fig. 6e). Increased counts of auxiliary T lymphocytes (*p* < 0.01; Fig. 6f) and cytotoxic T lymphocytes (*p* < 0.01; Fig. 6g) and decreased counts of B lymphocytes (*p* < 0.01; Fig. 6h) were observed in mice that received fecal microbiota from either COPD group. Overall, a higher proportion and number of T lymphocytes and lower proportion and number of B lymphocytes were observed in recipient mice that received fecal microbiota from COPD patients.

**Fig. 7:**
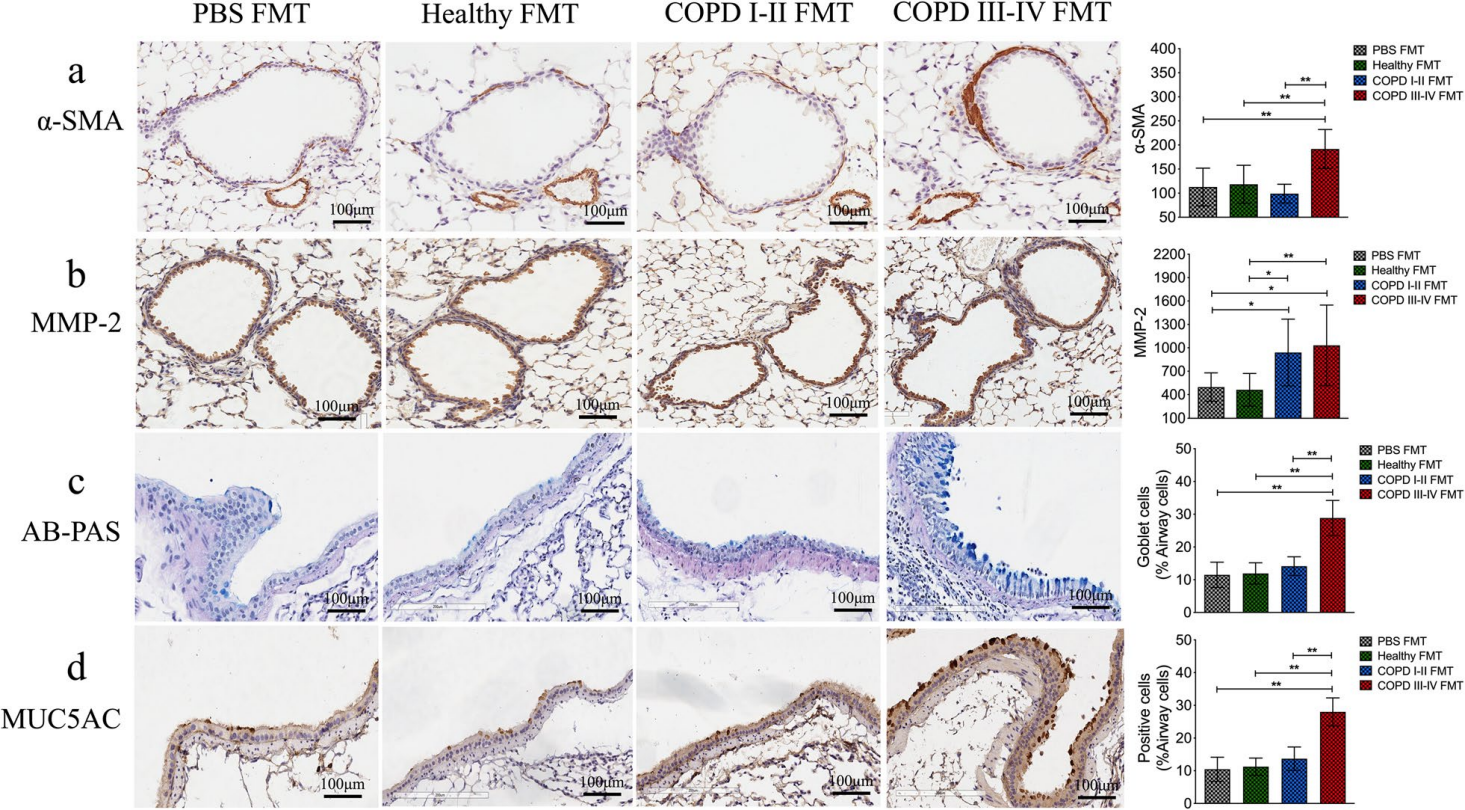
Intestinal microbiota from patients with COPD induce airway remodeling and mucus hypersecretion in the lungs of recipient mice. **a** Quantitative analysis showed that the integrative optical density (IOD) value of α-SMA was significantly higher in the COPD III–IV FMT group than the other group. **b** The IOD value of matrix metalloproteinase 2 (MMP-2) increased both in the COPD I–II and COPD III–IV FMT groups.** c** and** d** Immunohistochemistry showed higher goblet cells and MUC5AC levels in airway epithelial cells in the COPD III–IV FMT mouse group than in the other three group. Scale bars: 100 μm. Significance was determined by ANOVA and *p* values were corrected using the Bonferroni method. Results are means ± SD, n = 5 mice. *p < 0.05, **p < 0.01

We also assayed three key proteins to assess the activation of airway remodeling and mucus hypersecretion in the lung. Higher levels of α smooth-muscle actin were observed in the COPD III–IV FMT mouse group (*p* < 0.01, Fig. 7a) and expression of matrix metalloproteinase 2 around airways was higher in both COPD FMT mouse groups (*p* < 0.05 or 0.01, Fig. 7b). Immunohistochemistry showed that intestinal microbiota from patients with COPD III–IV promoted mucus hypersecretion (AB-PAS and MUC5AC, *p* < 0.01, Fig. 7c, d) in recipient mice. These results show that gut microbiota from COPD patients induced a greater pulmonary inflammatory response than microbiota from healthy controls.

### Intestinal microbiota accelerates COPD development in mice

**Fig. 8:**
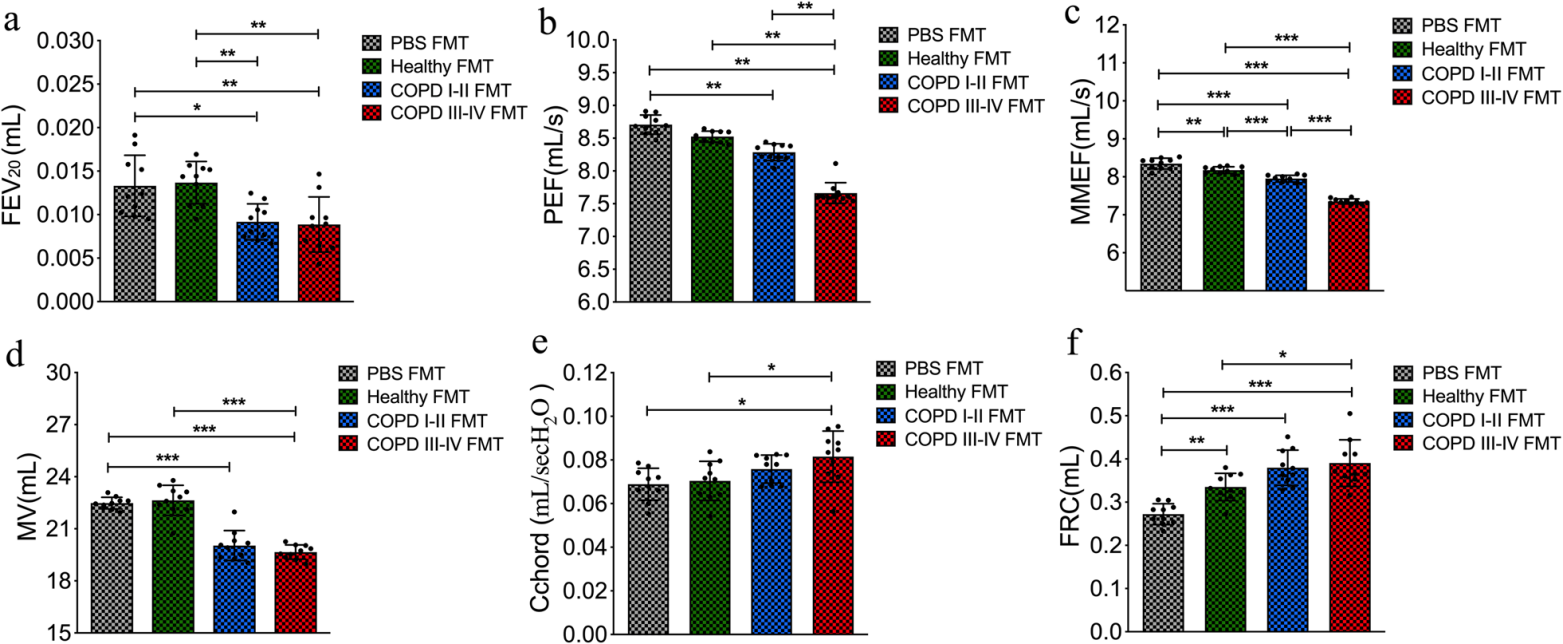
After 20 weeks of exposure, intestinal microbiota from patients with COPD accelerates declines in lung function in recipient mice. **a**–**d** After 20 weeks of exposure, the forced expiratory volume in 20 s (FEV_20_), MMEF (mean mid expiratory flow), minute ventilation volume (MV) and peak expiratory flow (PEF) were significantly lower in the mouse recipients of fecal microbiota from COPD I–II to III–IV patients than from controls. **e**, **f** The Cchord (chord compliance, between 0 and 10 cm H_2_0) and FRC (functional residual capacity) were significantly higher in the mouse recipients of fecal microbiota from COPD I–II to III–IV patients than from controls. Significance was determined by ANOVA and *p* values were corrected using the Bonferroni method. Results are expressed as means ± SD, n = 10 mice. *p < 0.05, **p < 0.01, ***p < 0.001

**Fig. 9:**
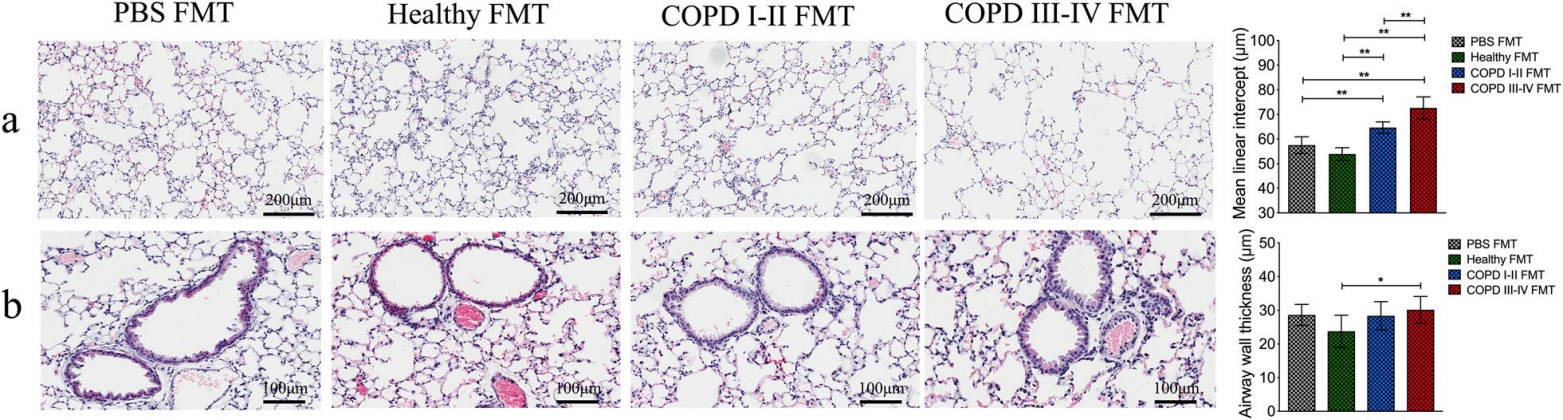
After 20 weeks of exposure, intestinal microbiota from patients with COPD accelerates emphysematous changes in recipient mice. **a** Lung sections stained with hematoxylin and eosin showing that mouse recipients of fecal microbiota from COPD patients developed significant airspace enlargement after 20 weeks of exposure to smoke from biomass fuel. **b** After 20 weeks of smoke exposure, the small airway wall was significantly thicker in the mice that received fecal microbiota transplantation (FMT) from III to IV subjects than in mice that received FMT from healthy controls. Scale bar: 100 or 200 μm. Significance was determined by ANOVA and *p* values were corrected using the Bonferroni method. Results are expressed as means ± SD, n = 5 mice. *p < 0.05, **p < 0.01

To further assess whether gut microbiota dysbiosis is a potential causal factor in the progression of COPD, we performed fecal microbiota transplantation in mice during biomass smoke exposure over 20 weeks. Additional file 3: Fig. S3 show the inhalation chamber and the size distributions of particulates inhaled by the mice during exposure. Additional file 4: Table S4 lists the concentrations of oxygen, carbon monoxide, nitrogen oxides, and sulfur dioxide during the exposures. After 20 weeks of exposure, the PEF, FEV_20_, MMEF and MV were significantly lower in the mouse recipients of fecal microbiota from COPD I–II and III–IV patients, while Cchord and FRC were significantly higher (*p* < 0.05, *p* < 0.01 or 0.001, Fig. 8a–f). Histological analysis showed that the mean linear intercept was significantly larger in mice that received fecal microbiota from COPD patients than in mice from the PBS and healthy controls groups (*p* < 0.01, Fig. 9a). Bronchial walls were thicker in the lungs of mice from the COPD III–IV FMT group (*p* < 0.05, Fig. 9b). Accelerated declines in lung function and emphysematous changes were observed in the mouse recipients of fecal microbiota from COPD patients during biomass smoke exposure.

**Fig. 10:**
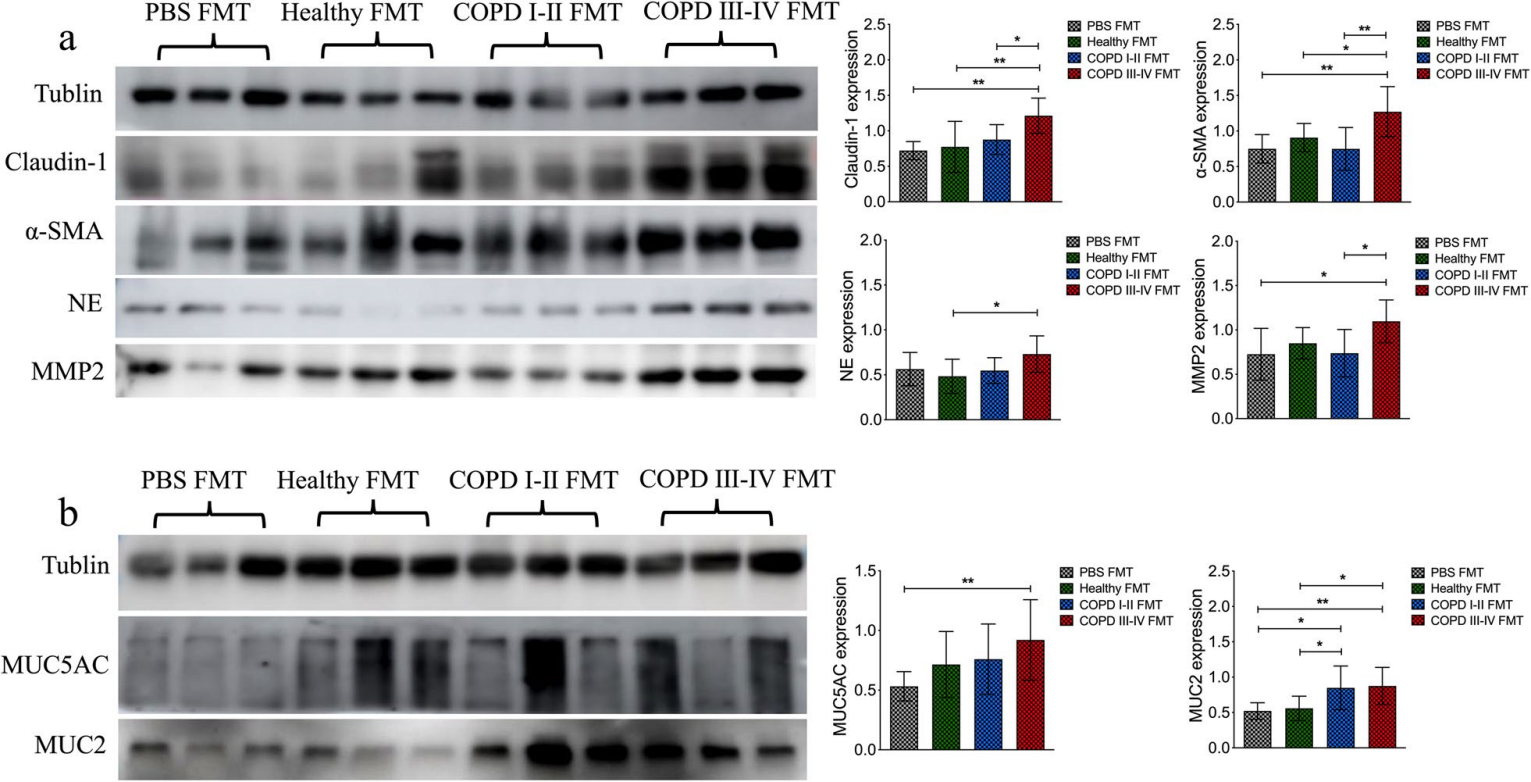
After 20 weeks of exposure, intestinal microbiota from patients with COPD induced airway remodeling and mucus hypersecretion in recipient mice. **a** Western blots of lung sections show that the expression levels of claudin 1, α smooth-muscle actin (α-SMA), neutrophil elastase (NE), and matrix metalloproteinase 2 (MMP-2) increased markedly in the COPD III–IV group in comparison with the healthy controls and COPD I–II fecal microbiota transplantation mice. **b** Representative western blots showing increased MUC5AC in the lungs of mice that received fecal microbiota transplantation from COPD III–IV patients. Higher levels of MUC2 were observed in both COPD FMT mouse groups. Significance was determined by ANOVA, and *p* values were corrected using the Bonferroni method. Results are expressed as mean ± SD, n = 10 mice. *p < 0.05, **p < 0.01

After 20 weeks of biomass smoke exposure, higher levels of the activation of airway remodeling and mucus hypersecretion-associated proteins were observed in mice that received fecal microbiota from COPD patients. Higher levels of claudin 1, α-SMA, NE and MMP-2 were observed in the COPD III–IV FMT mouse group (*p* < 0.01 or 0.01, Fig. 10a). Furthermore, higher levels of MUC5AC were observed in the COPD III–IV FMT mouse group and expression of MUC2 was higher in both COPD FMT mouse groups (*p* < 0.01 or 0.01, Fig. 10b).

## Discussion

To date, only a few studies have reported a direct association between the gut microbiome and COPD, and epidemiological evidence is lacking [31, 32]. We found that the gut microbiome of COPD patients was characterized by a distinct overall microbial diversity and composition and lower levels of SCFAs, especially in patients with lower pulmonary function (COPD III–IV). As the subjects were from a relatively restricted environment, the differences in their diets were small, limiting variations in the gut microbiome [33]. Our work provides the first direct evidence that altered gut microbiota in COPD patients is associated with airway inflammation and accelerated COPD progression in mice.

Dysbiosis in the gut microbiota has been linked to alterations in immune responses and to the development of lung diseases such as acute respiratory distress syndrome, pneumonia, and childhood asthma [34–36]. Recent research results show that faecal microbiome and metabolome of COPD patients are distinct from those of healthy individuals [31]. Another study found that gut microbiota composition significantly affects cigarette smoke-induced mouse COPD development, and a commensal bacterium *Parabacteroides goldsteinii* was shown to ameliorate COPD [32]. Our mouse experiments offer further support for a viewpoint of pathogenic links between microbiota and the gut-lung axis in COPD. We have confirmed the gut microbiota composition in COPD, when transplanted, causes airway inflammation. Furthermore, intestinal microbiota from COPD patients induced mucus hypersecretion and accelerated lung function decline and emphysematous changes in mouse lungs.

Chronic sterile inflammation is central to the pathobiology of obesity and obesity-related complications [37, 38], and previous research supports dysbiosis of Bacteroidetes and Firmicutes as a potential trigger of inflammation [39, 40]. Our results found that the relative proportion of Bacteroidetes was lower and that of Firmicutes was higher in COPD group when compare to healthy control, consistent with reports from studies of obesity and diabetes. At the family level, we observed differing relative abundances between groups for *Fusobacteriaceae*, *Prevotellaceae*, and *Bacteroidaceae*. *Prevotella* were enriched in the stool of COPD patients, which were previously identified as highly enriched in the gut microbiota of patients with rheumatoid arthritis [41]. Colonization with *Prevotella* was shown to exacerbate epithelial inflammation and reduce body weight in mice [42]; we observed a similar reduction in body weight and higher pulmonary inflammation in mice colonized with gut microbiota from COPD III to IV patients. Thus, *Prevotella* may be involved in COPD, perhaps by triggering the inflammatory response somehow. Individual bacterial species should be monocolonized in mice for a systematic analysis of host lung pathology/immune responses to colonization, further individual bacterial transfer experiments may be beneficial to clarify the mechanisms underlying the effect of gut microbiota in COPD pathogenesis.

In this study, the levels of SCFAs in stool from COPD III–IV patients were significantly lower than in stool from the other groups. This seems to be consistent with our previous research, that is gut microbial dysbiosis and lower levels of short-chain fatty acids were observed ​in a particulate matter-induced rat COPD model [27]. SCFAs have anti-inflammatory properties, improve gut barrier function and reduce intestinal bacterial translocation in the host. SCFAs are associated with reduced risk of various conditions, including COPD, irritable bowel disease, allergic asthma, and diabetes [43–46]. The potential of SCFAs to protect against lung inflammation and emphysema has been demonstrated in animal models [47, 48]. Hence, increasing SCFAs intake in COPD may confer a positive effect, as previously suggested [43].

The gut microbiota composition in COPD, when transplanted, causes COPD-like systemic and airway inflammation. CD3 + CD4 + and CD3 + CD8 + T cells were present in higher proportions in both COPD mouse groups. A growing body of evidence suggests that adaptive immune reactions are involved in the pathogenesis of COPD, especially abnormalities in the number and function of CD4 + and CD8 + T cell [49, 50]. Airway inflammation is a feature of COPD and is present in both the large and small airways. Our results demonstrate that intestinal microbiota from COPD patients caused lung inflammation and airway mucus hypersecretion in mice. Numerous studies have shown that mucus hypersecretion is a key factor in the development of COPD and could permanently damage the pulmonary function [51]. Furthermore, mucus hypersecretion in the large and small airways is a consistent feature in a murine model of COPD [28]. We also looked at the effects of gut microbiota on airway remodeling-associated proteins, since the link between increased airway remodeling and COPD is well-established [2].

Our study had several limitations. First, we surveyed the gut microbiota from healthy controls and COPD patients cross-sectionally. In the absence of longitudinal or interventional study designs, it is difficult to ascertain whether gut microbiota dysbiosis is a cause or a consequence of COPD. Second, there was significant difference in smoking history between the COPD patients and healthy controls in our cohorts, hence smoking can be a confounding factor in our study. Inclusion of an additional healthy control subgroup with matched smoking status both for gut microbiota profiling and fecal transplantation experiment is needed to better address this problem. Third, the COPD patients enrolled in this study were mixed with cigarette smoke exposure and biofuel exposure which may have an impact on research results. Fourth, the gut microbiota represents a large community of microorganisms, and each bacterial species should be monocolonized in mice for a systematic analysis of host lung pathology/immune responses to colonization. Lastly, in the mouse model of microbiome depletion by administration of antibiotics, lung microbiome will also be reduced along with gut microbiome. How the lung microbiome depletion influences the development of COPD are largely unknow.

## Conclusion

In summary, we present evidence for dysbiosis in the gut microbiota of COPD patients along with reduced levels of SCFAs and establish a potential link between gut microbiota dysbiosis, airway inflammation, and COPD progression. The role of the gut microbiome in the development of COPD should draw considerable attention.

## Supplementary Information

**Additional file 1: Fig. S1.** Phylum percent relative abundances. To determine the response of the host microbiome after receiving human fecal transplants, we analyzed the taxonomical community structure of the microbiome in mice fecal samples from the PBS FMT group, healthy FMT group, COPD I–II FMT group, and COPD III–IV FMT group. At the phylum level, all samples from the PBS FMT group, healthy FMT group, COPD I–II FMT group, and COPD III–IV FMT group contained four major bacterial phyla (%): Bacteroidetes, 36.84 (26.06, 45.97), 32.13 (23.29, 35.10), 33.05 (19.60, 37.07), and 29.77 (23.90, 32.58), respectively; Firmicutes, 40.33 (26.12, 50.17), 49.88 (44.50, 53.56), 53.60 (47.35, 68.73), and 54.26 (45.75, 57.32), respectively; Proteobacteria, 8.51 (5.20, 13.30), 3.30 (1.74, 7.29), 1.04 (0.49, 5.23), and 3.24 (1.06, 8.83), respectively; Actinobacteria, 0.06 (0.04, 1.42), 9.97 (3.33, 11.77), 7.11 (4.52, 10.32), and 4.49 (2.98, 8.92), respectively. Relative abundances of bacterial phyla differed (*p* < 0.05 or *p* < 0.01) between the PBS FMT group and fecal transplants group in Actinobacteria, Firmicutes, and Proteobacteria. There was no significant difference between the healthy FMT group, COPD I–II FMT group, and COPD III–IV FMT group. Results are expressed as the mean; n = 10 mice. Significance was determined by ANOVA and by Kruskal-Wallis test, and *p* values were corrected using the Bonferroni method.

**Additional file 2: Fig. S2.** Microbial abundance was measured in feces from human subjects and compared with microbial abundance in feces from recipient mice. At the phylum level, inoculum from healthy subjects, COPD I–II subjects, and COPD III–IV subjects contained four major bacterial phyla (%): Bacteroidetes, 54.50, 51.48 and 43.43, respectively; Firmicutes, 38.50, 39.04 and 46.96, respectively; Proteobacteria, 3.67, 6.22 and 7.53, respectively; Actinobacteria, 0.69, 1.08 and 0.65, respectively. As shown below, the characteristics of the murine gut microbial community post-fecal transplant were not identical to those profiled originally in the human-derived sample (A–C). Results are expressed as the mean.

**Additional file 3: Fig. S3.** The biomass smoke exposure system and particle size distributions during exposure. (a) All mice were exposed to biomass smoke in inhalation chamber systems. (b) Particulate matter concentrations and particle size distributions during biomass smoke exposure. Boxes and the inside line represent means ± SD for particulate matter with an aerodynamic diameter < 2.5 μm (PM2.5).

**Additional file 4: Supplementary table 1-4. Supplementary table 1.** Relative abundance of most abundant phyla (%) observed in stool samples collected in healthy control group and COPD group. **Supplementary table 2.** Relative abundance of most abundant family (%) observed in stool samples collected in healthy control group and COPD group. **Supplementary table 3.** The viability of bacteria in the inoculum in different microbial growth medium (CFU/ml). **Supplementary table 4.** Concentrations of particulate matter (PM) and gaseous pollutants measured during exposure.

**Additional file 5:** Ethics approval and consent to participate.

**Additional file 6:** Animal ethics approval.

## Data Availability

The raw sequencing data were deposited in the Sequence Read Archive database under accession number PRJNA606975. The R codes and detailed instruction for the enterotype analysis in this manuscript are now provided in GitHub (https://github.com/yzhaowei/2020_COPD_enterotypes/).

## Abbreviations

COPD: Chronic obstructive pulmonary disease
IL: Interleukin
PBS: Phosphate-buffered saline
FMT: Fecal microbiota transplantation
SCFA: Short-chain fatty acid
