# Supplementary figures and images for: Gut microbiota dysbiosis contributes to the development of chronic obstructive pulmonary disease
Source: Respir Res. 2021 Oct 25;22:274. doi: 10.1186/s12931-021-01872-z (PMC8543848; doi:10.1186/s12931-021-01872-z)

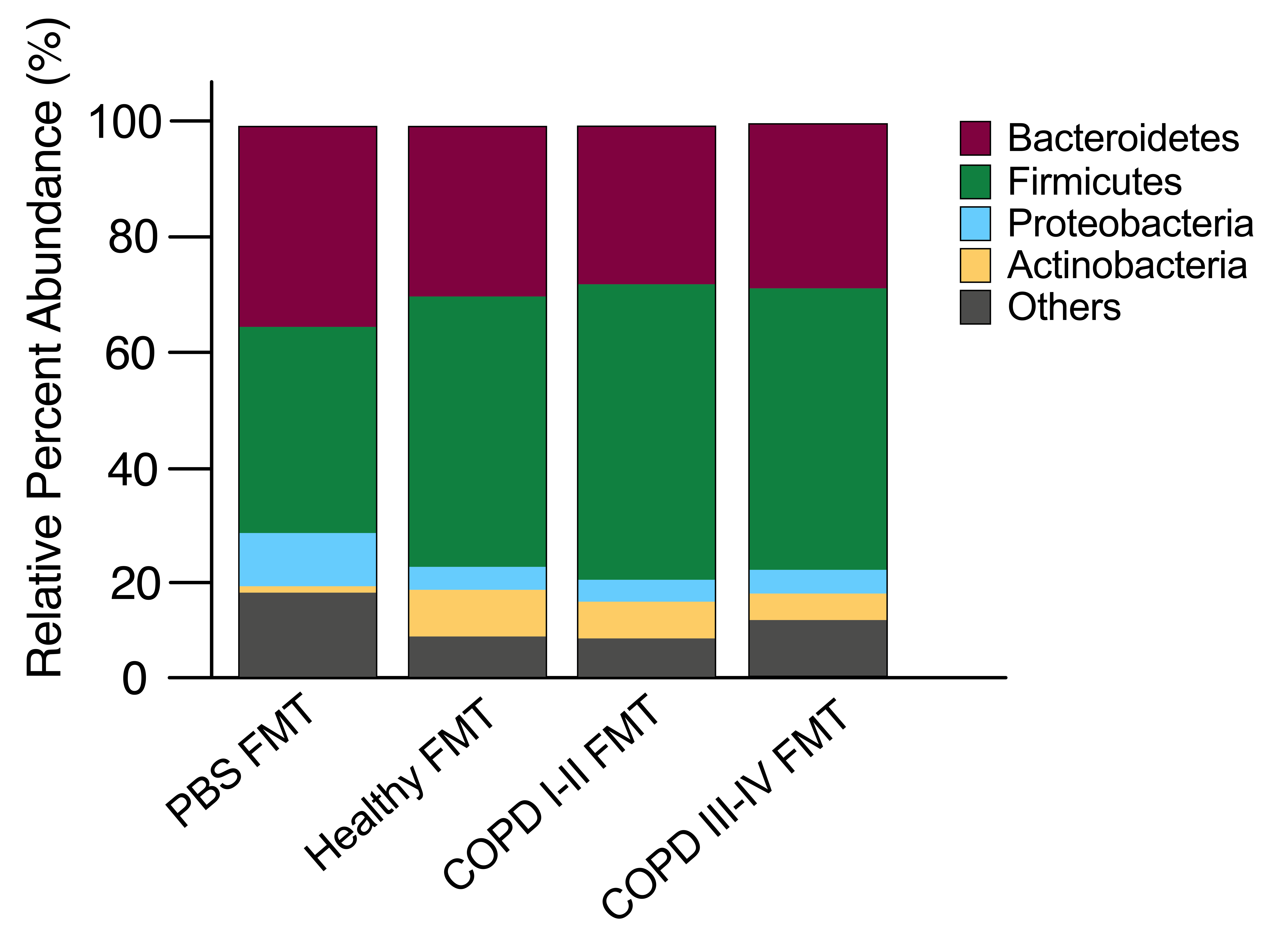

Supplement: Supplementary file 1 — Additional file 1: Fig. S1. Phylum percent relative abundances. To determine the response of the host microbiome after receiving human fecal transplants, we analyzed the taxonomical community structure of the microbiome in mice fecal samples from the PBS FMT group, healthy FMT group, COPD I–II FMT group, and COPD III–IV FMT group. At the phylum level, all samples from the PBS FMT group, healthy FMT group, COPD I–II FMT group, and COPD III–IV FMT group contained four major bacterial phyla (%): Bacteroidetes, 36.84 (26.06, 45.97), 32.13 (23.29, 35.10), 33.05 (19.60, 37.07), and 29.77 (23.90, 32.58), respectively; Firmicutes, 40.33 (26.12, 50.17), 49.88 (44.50, 53.56), 53.60 (47.35, 68.73), and 54.26 (45.75, 57.32), respectively; Proteobacteria, 8.51 (5.20, 13.30), 3.30 (1.74, 7.29), 1.04 (0.49, 5.23), and 3.24 (1.06, 8.83), respectively; Actinobacteria, 0.06 (0.04, 1.42), 9.97 (3.33, 11.77), 7.11 (4.52, 10.32), and 4.49 (2.98, 8.92), respectively. Relative abundances of bacterial phyla differed (p < 0.05 or p < 0.01) between the PBS FMT group and fecal transplants group in Actinobacteria, Firmicutes, and Proteobacteria. There was no significant difference between the healthy FMT group, COPD I–II FMT group, and COPD III–IV FMT group. Results are expressed as the mean; n = 10 mice. Significance was determined by ANOVA and by Kruskal-Wallis test, and p values were corrected using the Bonferroni method. [file 12931_2021_1872_MOESM1_ESM.tiff]

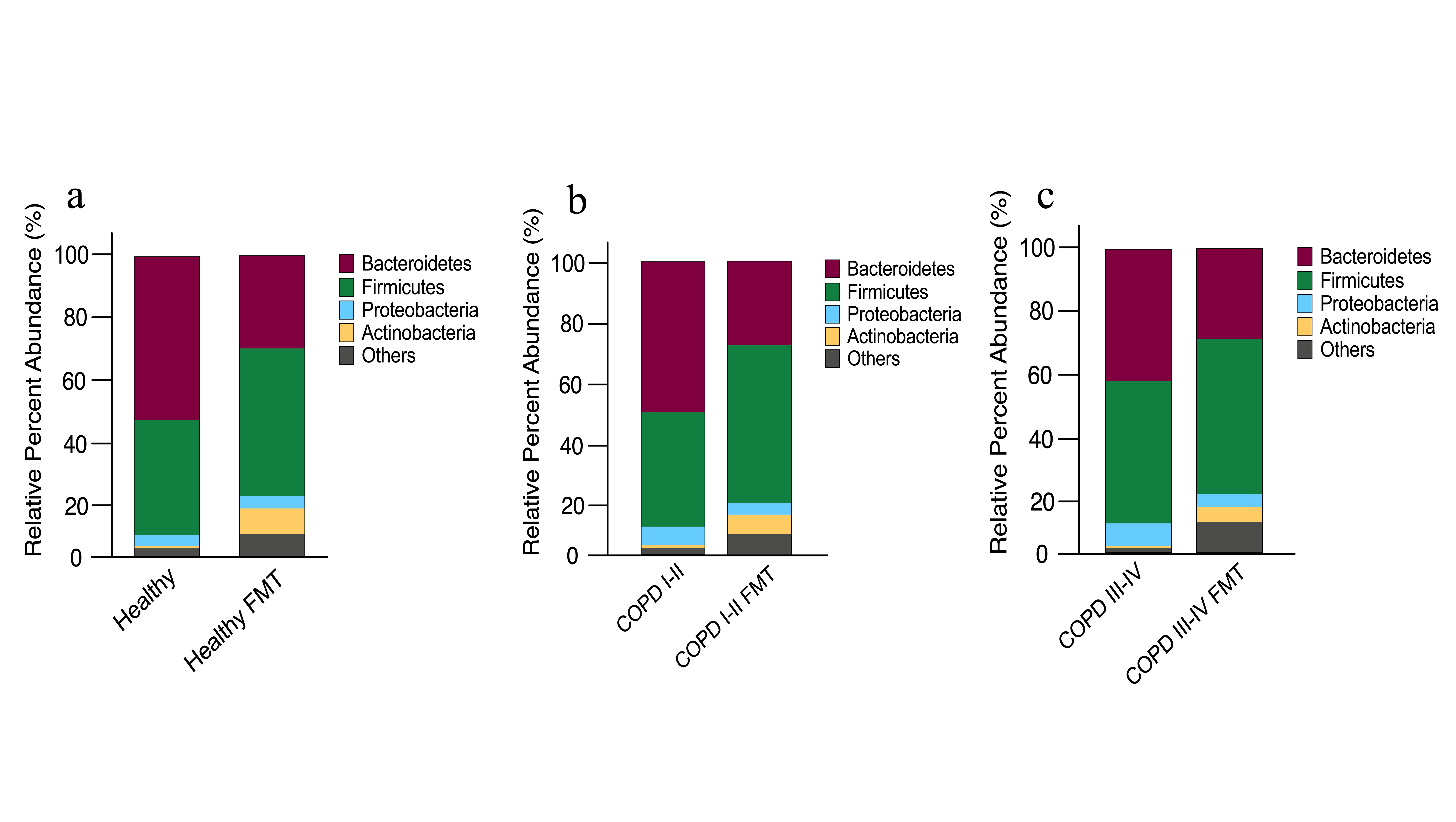

Supplement: Supplementary file 2 — Additional file 2: Fig. S2. Microbial abundance was measured in feces from human subjects and compared with microbial abundance in feces from recipient mice. At the phylum level, inoculum from healthy subjects, COPD I–II subjects, and COPD III–IV subjects contained four major bacterial phyla (%): Bacteroidetes, 54.50, 51.48 and 43.43, respectively; Firmicutes, 38.50, 39.04 and 46.96, respectively; Proteobacteria, 3.67, 6.22 and 7.53, respectively; Actinobacteria, 0.69, 1.08 and 0.65, respectively. As shown below, the characteristics of the murine gut microbial community post-fecal transplant were not identical to those profiled originally in the human-derived sample (A–C). Results are expressed as the mean. [file 12931_2021_1872_MOESM2_ESM.tiff]

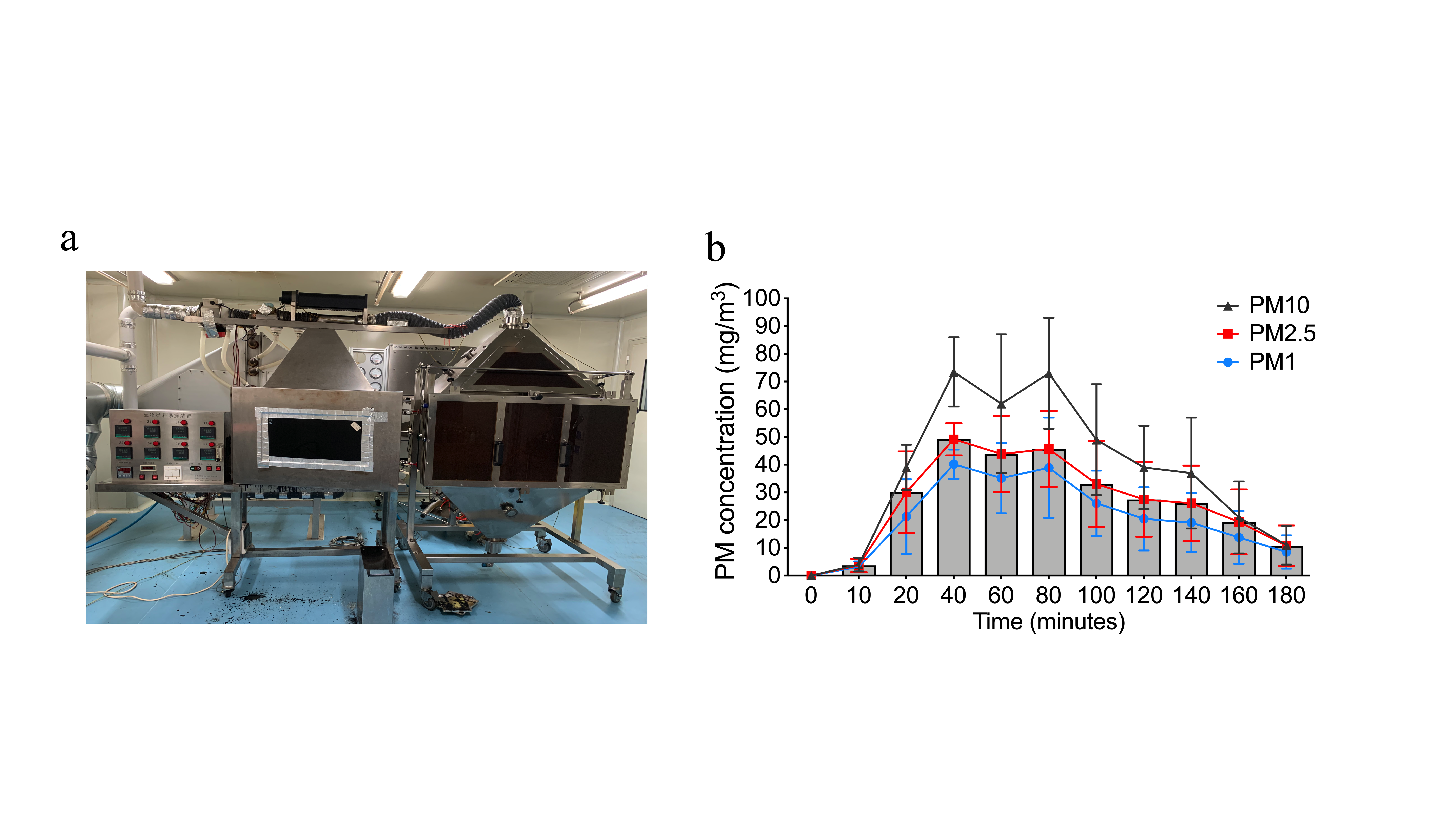

Supplement: Supplementary file 3 — Additional file 3: Fig. S3. The biomass smoke exposure system and particle size distributions during exposure. (a) All mice were exposed to biomass smoke in inhalation chamber systems. (b) Particulate matter concentrations and particle size distributions during biomass smoke exposure. Boxes and the inside line represent means ± SD for particulate matter with an aerodynamic diameter < 2.5 μm (PM2.5). [file 12931_2021_1872_MOESM3_ESM.tiff]
