# Supplementary material for: Gut microbiota dysbiosis contributes to the development of chronic obstructive pulmonary disease
Source: Respir Res. 2021 Oct 25;22:274. doi: 10.1186/s12931-021-01872-z (PMC8543848; doi:10.1186/s12931-021-01872-z)
Supplement: Supplementary file 4 — Additional file 4: Supplementary table 1-4. Supplementary table 1. Relative abundance of most abundant phyla (%) observed in stool samples collected in healthy control group and COPD group. Supplementary table 2. Relative abundance of most abundant family (%) observed in stool samples collected in healthy control group and COPD group. Supplementary table 3. The viability of bacteria in the inoculum in different microbial growth medium (CFU/ml). Supplementary table 4. Concentrations of particulate matter (PM) and gaseous pollutants measured during exposure. [file 12931_2021_1872_MOESM4_ESM.docx]

**Additional Table**

**Additional table S1. Relative abundance of most abundant phyla (%) observed in stool samples collected in healthy control group and COPD group.**

| **Phylum (%)** | **Healthy group** | **COPD I-II group** | **COPD III-IV group** |
| --- | --- | --- | --- |
| Bacteroidetes | 52.23 (41.18,61.01) | 52.99 (45.21,62.57) | 41.86 (34.02,56.82) |
| Firmicutes | 40.31 (29.89,52.33) | 37.81 (29.48,45.90) | 43.66 (35.81,55.01) |
| Proteobacteria | 3.07 (2.09,5.65) | 3.03 (1.89,7.25) | 4.92 (2.31,8.33) |
| Actinobacteria | 0.20 (0.09,0.62) | 0.33 (0.12,0.62) | 0.23 (0.11,0.54) |
| Others | 0.51 (0.09,2.11) | 0.44 (0.08,2.56) | 0.11 (0.04,1.71) |

Legend: Data are shown as median (interquartile range).

**Additional table S2. Relative abundance of most abundant family (%) observed in stool samples collected in healthy control group and COPD group.**

| **Family (%)** | **Healthy group** | **COPD I-II group** | **COPD III-IV group** |
| --- | --- | --- | --- |
| Bacteroidaceae | 38.98 (17.03,50.06) | 27.13 (10.28,47.34) | 21.61 (8.06,38.64) |
| Porphyromonadaceae | 0.86 (0.42, 1.39) | 0.72 (0.44,1.31) | 0.79 (0.30,1.46) |
| Prevotellaceae | 0.02 (0.00,17.49) | 2.95 (0.01,41.41) | 0.46 (0.00,26.93) |
| Rikenellaceae | 0.21 (0.01,0.53) | 0.25 (0.04,0.74) | 0.19 (0.02,0.69) |
| Odoribacteraceae | 0.23 (0.01,0.52) | 0.18 (0.02,0.52) | 0.19 (0.00,0.65) |
| Firmicutes;f__ | 2.00 (0.60,3.55) | 2.25 (0.84,5.39) | 2.85 (0.72,6.42) |
| Clostridiaceae | 0.22 (0.09,1.10) | 0.33 (0.11,0.92) | 0.51 (0.16,1.22) |
| Lachnospiracea | 13.09 (7.99,18.80) | 9.88 (7.29,15.94) | 13.55 (6.76,17.97) |
| Peptostreptococcaceae | 0.04 (0.01,0.15) | 0.04 (0.01,0.15) | 0.04 (0.01,0.12) |
| Ruminococcaceae | 10.64 (5.05,15.21) | 12.34 (7.06,17.74) | 12.10 (7.03,17.66) |
| Veillonellaceae | 9.44 (4.77,16.16) | 7.44 (4.76,10.16) | 7.49 (3.92,15.34) |
| Fusobacteriaceae | 0.27 (0.01,1.96) | 0.06 (0.00,1.25) | 0.01 (0.00,0.05) |
| Alcaligenaceae | 1.24 (0.55,1.96) | 0.89 (0.48,1.40) | 0.75 (0.37,1.42) |
| Desulfovibrion-aceae | 0.20 (0.04,0.44) | 0.26 (0.11,0.50) | 0.19 (0.02,0.65) |
| Enterobacteriaceae | 0.81 (0.21,2.20) | 1.03 (0.24,2.95) | 1.43 (0.08,5.67) |

Legend: Data are shown as median (interquartile range).

**Additional table S3. The viability of bacteria in the inoculum in different microbial growth medium (CFU/ml).**

| **Microbial growth medium** | **Healthy group** | **COPD I-II group** | **COPD III-IV group** |
| --- | --- | --- | --- |
| Eosin-Methylene blue agar | 3.4x 10^6^ | 1.2x 10^7^ | 8.0x 10^6^ |
| Luria-Bertani agar | 4.8x 10^6^ | 4.0x 10^7^ | 6.6x 10^6^ |
| Blood agar | 1.6 x 10^9^ | 7.8x 10^7^ | 6.4x 10^7^ |
| MacConkey agar | 4.0x 10^6^ | 1.3x 10^7^ | 6.0x 10^6^ |

CFU, Colony-forming units

**Additional table S4. Concentrations of particulate matter (PM) and gaseous pollutants measured during exposure**

| **Test items** | **Concentrations** |
| --- | --- |
| PM_10_ (mg/m^3^) | 37.1±26.1 |
| PM_2.5_ (mg/m^3^) | 26.3±16.6 |
| PM_1_ (mg/m^3^) | 20.6±13.7 |
| NO_1_ (ppm) | — |
| NO_X_ (ppm) | — |
| SO_2_ (ppm) | — |
| CO (ppm) | 51.3±42.3 |
| O_2_ (%) | 20.9 ± 0.04 |
| Humidity (%) | 60.1±5.6 |
| Temperature (℃) | 24.9±0.5 |

Legend: Values are shown as mean ± SD.
