# Supplementary material for: Gut microbiota dysbiosis contributes to the development of chronic obstructive pulmonary disease
Source: Respir Res. 2021 Oct 25;22:274. doi: 10.1186/s12931-021-01872-z (PMC8543848; doi:10.1186/s12931-021-01872-z)
Supplement: Supplementary file 5 — Additional file 5: Ethics approval and consent to participate. [file 12931_2021_1872_MOESM5_ESM.pdf]

广州医科大学附属第一医院科研项目审查伦理委员会

临床试验会议审批件

医科伦审 2017 第 21 号

项目名称	金康速力-乙酰半胱氨酸泡腾片长期规则治疗早期 COPD 的平行分组、随机双盲安慰剂对照多中心临床研究			
申办者	广州医科大学附属第一医院呼研所	项目负责人	冉丕鑫	
审查类别	会议审查（初审）			

表决结果					
	委员人数 11 人	出席人数 7 人	回避人数 0 人	弃权人数 0 人	
	同意	作必要的修正后同意	作必要的修正后重审	不同意	终止或暂停先前批准的试验
	4	3	0	0	0

结论	同意
审查意见	1、请就知情同意书中（研究背景和目的）的死因进行文献说明，增加科学性。<br>2、请明确受试者的益处及免费项目。<br>3、不建议扩大药品适应症。<br>4、知情同意书请认真修改，并附上说明书。

结合此前对该临床研究的伦理审查意见，现已对申办方提交的材料进行补充备案，同意批准“金康速力-乙酰半胱氨酸泡腾片长期规则治疗早期 COPD 的平行分组、随机双盲安慰剂对照多中心临床研究”的实施。

伦理委员会主任委员签字：[illegible]　日期：2017-8.22

广州医科大学附属第一医院医学伦理委员会（盖章）

（印章：广州医科大学附属第一医院 医学伦理委员会）

备注	
	1. 修改后同意/重审项目，应将修改后文件及时反馈伦理委员会，进行进一步审查。<br>2. 不同意/终止或暂停项目，批件发出 2 周内可向伦理委员会就有关事项做出解释或提出申诉。<br>3. 临床试验应严格按照本伦理委员会批准的文件执行。在试验实施过程中，如对试验方案、知情同意书等文件做任何修改，应及时向本伦理委员会提交变更申请，补充更新文件，经伦理委员会重新审查批准后，方可执行。<br>4. 发生严重不良事件及可能影响风险受益的任何事件和新信息须及时报告本伦理委员会。<br>5. 定期/年度跟踪审查项目，于到期后 1 周内提交试验进度情况报告。如有不依从/违背方案或暂停/提前终止的试验项目，应及时以书面文件告知本伦理委员会。试验结束后，须及时向伦理委员会提交结题报告。<br>6. 本批件有效期为 1 年（自批准之日起）。若在有效期内未启动项目，则本批件自动终止。<br>7. 本委员会依据 GCP 和国家法规以及 ICH-GCP 的要求操作。

审查日期：2017-05-04	审查地点：广州医科大学附属第一医院新大楼二十九楼会议室	
联系方式：广州市越秀区沿江西路 151 号（邮编 510120）		
电话：020-83062938	传真：020-83389471	联系人：余达加
