# Supplementary material for: Gut microbiota dysbiosis contributes to the development of chronic obstructive pulmonary disease
Source: Respir Res. 2021 Oct 25;22:274. doi: 10.1186/s12931-021-01872-z (PMC8543848; doi:10.1186/s12931-021-01872-z)
Supplement: Supplementary file 6 — Additional file 6: Animal ethics approval. [file 12931_2021_1872_MOESM6_ESM.pdf]

广州医科大学实验动物伦理委员会审查报告

Committee review of animal experiments in Guangzhou Medical University

项目名称<br>(Title)	肠-肺轴在慢阻肺发病机制中的作用和机制<br>The role of gut-lung axis in chronic obstructive pulmonary disease and its mechanism		
拟申请课题或资金来源<br>(Project sources)	中国国家自然科学基金<br>National Natural Science Foundation of China		
项目申请人<br>(Applicant)	李冰	受理编号<br>(Acceptance number)	GY2018-084
审查形式<br>(Auditing)	函审<br>Letter correspondence	审查时间<br>(Processing time)	2018.10.18

审查结果(Results of the review)：

该实验方案符合动物福利伦理要求，通过动物实验伦理审查，准予开展动物实验。

According to the rules of Committee on Animal Research and Ethics, this research project has been reviewed and approved to be appropriate and humane by institutional animal care and use committee.

广州医科大学实验动物伦理委员会

Institutional Animal Care and Use Committee of Guangzhou Medical University

签章(Signature)： [illegible]

时间(time)： 2018.10.18

扫描全能王 创建
